# A scoping review of system-level mechanisms to prevent children being in out-of-home care

**DOI:** 10.1093/bjsw/bcab213

**Published:** 2021-11-09

**Authors:** Lorna Stabler, Rhiannon Evans, Jonathan Scourfield, Fiona Morgan, Alison Weightman, Simone Willis, Lydia Searchfield, Mel Meindl, Sophie Wood, Ulugbek Nurmatov, Alison Kemp, Donald Forrester, Sarah L Brand

**Affiliations:** Children’s Social Care Research and Development Centre (CASCADE), Cardiff University, Cathays, CF10 3BD, UK; Centre for Development, Evaluation, Complexity and Implementation in Public Health Improvement (DECIPHer), Cardiff University, Cathays, CF10 3BD, UK; Children’s Social Care Research and Development Centre (CASCADE), Cardiff University, Cathays, CF10 3BD, UK; Public Health Wales, No. 2. Capital Quarter, Cardiff, CF10 4BZ, UK; Specialist Unit for Review Evidence, Cardiff University, Neuadd Meirionnydd, Cardiff, CF14 4YS, UK; Specialist Unit for Review Evidence, Cardiff University, Neuadd Meirionnydd, Cardiff, CF14 4YS, UK; Specialist Unit for Review Evidence, Cardiff University, Neuadd Meirionnydd, Cardiff, CF14 4YS, UK; Children’s Social Care Research and Development Centre (CASCADE), Cardiff University, Cathays, CF10 3BD, UK; Children’s Social Care Research and Development Centre (CASCADE), Cardiff University, Cathays, CF10 3BD, UK; School of Medicine, University Hospital Wales, Cardiff, CF14 4XN, UK; School of Medicine, University Hospital Wales, Cardiff, CF14 4XN, UK; Children’s Social Care Research and Development Centre (CASCADE), Cardiff University, Cathays, CF10 3BD, UK; NIHR Applied Research Collaboration South West Peninsula (PenARC), University of Exeter Medical School, Exeter, Devon, EX1 2LU, UK

**Keywords:** child maltreatment, child protection, intervention, scoping review, social work, state care

## Abstract

Identifying which approaches can effectively reduce the need for out-of-home care for children is critically important. Despite the proliferation of different interventions and approaches globally, evidence summaries on this topic are limited. This study is a scoping review using a realist framework to explore what research evidence exists about reducing the number of children and young people in care. Searches of databases and websites were used to identify studies evaluating intervention effect on at least one of the following outcomes: reduction in initial entry to care; increase in family reunification post care. Data extracted from papers included type of study, outcome, type and level of intervention, effect, mechanism and moderator, implementation issues and economic (EMMIE) considerations. Data were coded by: primary outcome; level of intervention (community, policy, organisation, family or child); and type of evidence, using the realist EMMIE framework. This is the first example of a scoping review on any topic using this framework. Evaluated interventions were grouped and analysed according to system-level mechanism. We present the spread of evidence across system-level mechanisms and an overview of how each system-level mechanism might reduce the number of children in care. Implications and gaps are identified.

## Teaser

There have been many ways attempted to reduce the number of children in care. It is difficult to know which, if any, have successfully achieved this aim, and for which children under which circumstances and in what way. One way of trying to find this out is to do a review of the literature. This review looked for published scientific papers that evaluated whether programmes that aim to reduce the number of children in care worked. The review grouped and summarised papers based on the main way in which the programmes they evaluated aimed to reduce numbers. This overcame the common problem in this literature of programmes that used similar approaches having a few different names. Looking at them based on approach rather than name gave richer information about each type of approach or ‘key mechanism’. In total, nine of these ‘key mechanisms’ were identified, and data are presented which shows how much evidence there is about if they work, how they might work, how they are implemented and cost implications of them.

## Introduction

Increasing numbers of children are in the English care system, rising from 50,900 in 1997 to 80,080 in 2020 (Department for Education, 2020). This trend is similar in Australia (Australian Institute of Health and Welfare, 2020), Germany and the Netherlands (Harder *et al.*, 2013), although a reversal of this trend seems to have been seen in the USA (Children’s Bureau, 2019). There are hypotheses but no consensus on the reasons for increases or decreases in the care population (Bywaters* et al.,* 2020).

Individuals who come into contact with the care system experience a range of adverse outcomes across their lives compared to the general population, including higher rates of psychological disorders and poorer educational attainment (Ford *et al.*, 2007; Trout *et al.*, 2008). Preventing the need for children and young people to enter state care is therefore a significant social, health and educational priority in many countries such as England (All Party Parliamentary Group for Children, 2017), Wales (Improving Outcomes for Children Advisory Group, 2019), New Zealand (Katz *et al.*, 2015), Australia (Commonwealth of Australia, 2009) and Norway (Skivenes, 2011).

There is some consensus around the need to prevent the risk factors for care entry (Department for Education, 2016; Family Rights Group, 2018). This follows the principles of the United Nations Convention on the Rights of the Child (1989) and the Children Act (UK, 1989), which emphasise the importance of a child being cared for by their parents, while acknowledging that for some children care entry will remain the safest and most appropriate course of action. It must also be acknowledged that part of the reason for rising out-of-home care rates over time must be practice decisions and professionals’ changing responses to risk, so child welfare organisations are implicated in the rising rates as well as change in how social problems manifest (Thomas, 2018).

Preventative interventions can operate across various socio-ecological intervention points, using different resources, and aim to address a wide range of issues (Mcleroy *et al.*, 1988). These can include: interpersonal interventions that focus on communication within the family (e.g. Intensive Family Preservation Services (Forrester *et al.*, 2016)); organisational interventions aimed at modifying social work practice and ethos (e.g. Signs of Safety (Turnell and Edwards, 1997)) and national policy strategies (e.g. Department for Education’s (2016) Putting Children First; Scotland’s Getting It Right For Every Child policy). To evaluate the impact of these interventions on outcomes at levels beyond the individual or family it is possible that population-level change might be evidenced—for example, rate of Child Protection referrals in an area, child well-being and parenting practices as reported in general population surveys.

The evidence base remains mixed and it is somewhat unclear how the use of state care might effectively be reduced. Understanding is limited about the transportability of intervention effects across heterogeneous social care systems, where different contextual characteristics may impede the replication of outcomes. For example, the US-evaluated Multi-Systematic Therapy has not replicated effects in the UK (Fonagy *et al.*, 2018).

This study maps and systematically draws together social care literature on the prevention of care entry or the increase of children returning, and remaining at home after a period in state care. This synthesis aims to illustrate where evidence may exist for the outcomes of relevant interventions, and how they work, for whom, under which circumstances. This article also presents an innovative way of conducting a scoping review, highlighting different types of evidence rather than just effectiveness data. This article is an overview of the data, and more detailed work explores the methodology (Brand *et al.*, 2019), and further synthesis of some of the findings (Stabler *et al.*, 2019).

### Scoping review

A scoping review broadly maps an area of interest. Scoping reviews have been identified as a useful methodology in social care to map out and categorise existing literature in a particular area (e.g. Bates and Coren, 2006). This scoping review maps the evidence on what works in reducing the number of children and young people in state care, identifying evidence clusters, gaps and uncertainties (Armstrong *et al.*, 2011). This scoping review focused on grouping interventions not by name but by ‘key system-level mechanism’—main intervention resource + the response it triggers from stakeholders to lead to the resulting outcome.

The review will lead to a suite of further research exploring findings in greater detail to understand the contexts important in enabling the identified mechanism to safely reduce the number of children in care. Nuanced analysis of context–mechanism–outcome chains underneath the relationship between each system-level mechanism and the outcome of safe care reduction will be undertaken in this future research.

Both peer-reviewed and grey literature were included as evidence may not always be published in peer-reviewed journals or indexed in academic databases. It was also important to capture innovation and work in progress that might not be peer reviewed yet (Adams *et al.*, 2016).

The review is concerned with the reduction in the number of children in care, while seeking the identification and support of those who do need to be removed from birth families. Preliminary searches were undertaken, and no existing scoping reviews were identified.

### Research questions

The scoping review addresses the following questions:

What is the nature and quantity of evidence for interventions that aim to:

- reduce the number of children and young people entering state care?
- increase and maintain the reunification of children and young people with their families following a period in out-of-home state care?

## Method

### Conceptual model

This scoping review adopts a realist approach to evidence mapping (Pawson and Tilley, 1997; Sharland and Taylor, 2006; Johnson *et al.*, 2015). Realist reviews approach searching and synthesising data from interventions in a way that seeks to unpack the mechanism of how complex programmes work (or why they fail) in specific contexts and settings (Pawson *et al.*, 2005). Rather than focusing on intervention effects, realist approaches consider the question of what works, for whom, in which circumstances, and in what way. In this way, the realist approach taken in the review has some principles in common with complex systems thinking, in that it is interested in the contextual contingencies of intervention effects (Fletcher *et al.*, 2016).

Evidence is not appraised or synthesised according to aggregate intervention effect sizes. Instead, to operationalise the realist approach, evidence is considered in relation to a composite assessment of measures prescribed by the realist effect, mechanism and moderator, implementation issues and economic (EMMIE) framework considerations (Johnson *et al.*, 2015), which supports the interrogation of a heterogeneous and complex evidence base.

The EMMIE framework comprises five dimensions for evidence mapping according to the review questions:

- effect (E) of an intervention;
- mechanisms (M) through which an intervention is expected to have an effect;
- the contexts that moderate (M) if mechanisms will be activated to generate the intended effect;
- barriers and facilitators of implementation (I); and
- economic (E) cost–effectiveness (Johnson *et al.*, 2015).

These dimensions were developed as pragmatic and meaningful for the presentation of evidence for policy-makers and commissioners.

The framework has primarily been employed to assess existing reviews or in systematic reviews of primary evidence (Johnson *et al.*, 2017; Sidebottom *et al.*, 2017). This is the first use of the EMMIE framework with a scoping review. We believe it offers a practical and comprehensive approach that captures the diverse and often rich data within the children’s social care literature, which is often overlooked by other methods of scoping review.

### Design

This scoping review methodology is structured and reported in accordance with Arksey and O’Malley’s methodological guidance (Arksey and O’Malley, 2005) and Levac *et al.*’s (2010) methodological enhancement. There are six stages: (1) identify the research question(s); (2) identify relevant studies; (3) study selection; (4) chart the data; (5) collate, summarise and report results and (6) consult with relevant stakeholders. Protocol components have been cross referenced with the Preferred Reporting Items for Systematic review and Meta-Analysis Protocols checklist to ensure completeness (Moher *et al.*, 2015). Further details on the method are published in the protocol, particularly in relation to the context of the study and research question identification (Brand *et al.*, 2019).

### Eligibility criteria

Full eligibility criteria were published prior to the review (Brand *et al.*, 2019). The eligibility criteria were developed in accordance with the Population, Intervention, Comparator, and Outcome (PICO) framework (Moher *et al.*, 2015). An additional **evaluation** (E) criterion was included to incorporate the EMMIE framework (see Supplementary File S1 for PICO(E) descriptors). To meet the aims of the scoping review, studies had to provide quantitative evidence of effect (first E in EMMIE).

To maximise the relevance of results to the UK, inclusion was limited to the following countries: UK, Ireland, USA, Canada, Australia, New Zealand, France, Germany, Sweden, Finland, Norway, Denmark and Netherlands. While there are differences in the legal and social frameworks, research from these countries was deemed more applicable because they all have well-established state care infrastructure, which is essential to the outcomes of interest.

Studies were included if the data were collected from 1991 onwards to coincide with the implementation in the UK of the Children Act 1989. Inclusion decisions took into account study design, population, intervention, outcome, evaluation and ability to disaggregate data. Only studies where full texts were available in English were included. This was a pragmatic decision due to resource constraints.

The overall outcome of reducing the number of children in care included two outcomes: care entry and reunification from care. The outcome of care entry refers to children entering care for more than 24 h either through a voluntary care order or a court order. The outcome of reunification from care refers to children returning to live with their parent(s) after a period of time in care, and remaining with family rather than returning to care after a period of being returned home.

### Information sources

Eighteen databases were searched to identify relevant peer reviewed literature. Grey literature was identified through a large range of online resources (see Supplementary File S2 for databases, websites and search terms). It was not feasible to search all relevant websites, therefore grey literature searches focused on sites recommended by key policy and practice stakeholders (e.g. What Works Centre for Children’s Social Care; Department for Education).

### Study selection

Ten percent of studies were screened independently by all members of the review team to refine the inclusion criteria and develop consistency in approach and decision making. Following this, study title and abstracts were independently screened against the inclusion criteria by two reviewers. A safety-first approach was adopted—if one reviewer included a title/abstract, then the full text was examined. Exclusion reasons were recorded at full text stage. Discrepancies were resolved in discussion by consensus and, where this was not possible, a third reviewer arbitrated.

### Data extraction

All included studies were imported to NVivo 12 (QSR, 2012). Data were extracted across the following domains: Outcome (care entry, reunification); Intervention type (intervention activities/resource); Socio-ecological domain of intervention (community level, policy level, organisational level, family level or interpersonal (child) level); EMMIE dimension. Data study characteristics (authors, year of publication, country, study design, target population (e.g. family, children, young people, social worker)) were extracted into an Excel worksheet.

Due to the complexity of the data extraction, four reviewers independently extracted outcome, EMMIE, socio-ecological and intervention data and discussed decisions as a group for 10 percent of studies. Data were extracted from the remaining studies independently by three reviewers, with a fourth reviewer to resolve issues. Regular meetings to discuss emerging issues ensured ongoing consistency. Study characteristics were extracted by additional research and administrative staff as available and checked by a member of the review team. Data within each paper were coded in NVivo 12. A hierarchical coding tree was developed according to domains of interest with a subset of studies and was refined in discussion with the whole team. Memos were generated to aid reviewer reflexivity.

### Risk of bias

Scoping reviews intend to map the concepts underpinning a research area and the main sources and types of evidence available (Jolley *et al.*, 2017), rather than assess the quality of individual studies. In line with prescribed scoping review methodology, no comment is made on the strength or quality of these studies as that is beyond the parameters of a scoping review. Instead, the review identifies where there may be enough evidence to carry out further synthesis of data, including quality appraisal. This is a common limitation of scoping reviews, which are not designed to assess the quality of studies, and are often conducted as a precursor to a systematic review in a more focused, narrow area (Munn *et al.*, 2018). While including studies of varying quality limits the ability to draw inferences on the effectiveness of an intervention, the mechanisms through which the intervention is thought to work are still of theoretical relevance for identifying which areas of intervention are explored in the literature. The purpose of this scoping review is to inform a suite of further primary and secondary research studies, which includes assessment of the quality of the evidence identified. For study characteristics such as sample size and study design of included studies see Supplementary File S3.

### Data analysis

Data analysis progressed in two steps:

1. Thematic analysis of intervention type to identify ‘key system-level mechanisms’ through with interventions were conceptualised to work.
2. Mapping and narrative descriptions of the evidence for each.

In this scoping review, interventions are grouped by system-level mechanism, and the other four dimensions of the EMMIE framework are reported numerically to identify where clusters of evidence exist in relation to each system-level mechanism.

#### Thematic analysis of intervention type: developing the coding structure

The thematic analysis was informed by pragmatic consideration of the most practical and useful way of summarising the nature of data available for use by research and practice. While some interventions in the studies shared a name, their related intervention activities varied. Descriptions of the actually delivered and implemented intervention resources were too often limited or absent. This made meaningfully grouping evidence extracted by intervention name and/or resources a significant challenge. For this reason, the decision was made to conduct a thematic analysis within and across each intervention type, identifying the ‘key system-level mechanism’ through which the intervention seemed to work, and grouping these interventions together. This thematic analysis was carried out iteratively by the three researchers (L.S., M.M. and S.W.) extracting data, with daily discussion about the inclusion criteria for each emergent grouping. The developing coding structure was checked in daily meetings between the three researchers and regular meetings with the senior researcher (S.L.B.), with uncertainties resolved by consensus. Weekly meetings with the senior researcher were used to further develop and group sources.

#### Summarising the evidence: mapping and describing

Evidence was summarised in two ways: a visual map and a narrative descriptive summary. For the visual map, the number of sources of data in each code was counted. As an example, if a code included five pieces of evidence from one source and one from another source that was counted as two sources of evidence for that code in the map. These numbers were used to create a visual map of the amount of evidence related to each of the EMMIE domains for the themes identified.

For the narrative summaries, a descriptive numerical summary analysis was undertaken (Levac *et al.*, 2010). Evidence coded in NVivo to each node in the code structure was summarised in a descriptive narrative of the nature of the evidence identified for each EMMIE category. Synthesis of data regarding intervention descriptions and resources was carried out through comparing and contrasting qualitative data in studies and refining categories through group discussion. A report detailing the findings of this review was peer reviewed, which resulted in further refining of categories. Note, some categories are similar, but each aims to identify a distinct ‘key system-level mechanism’ through grouping interventions that use the same main resource/s to trigger a similar response in participants to achieve a desired outcome.

### Stakeholder consultation

Consultation was carried out through meetings with policy and practice stakeholders before the review to identify the area of interest and refine the question, during the review to gain feedback on developing findings and categorisation of intervention types, and after the review for feedback on the findings and dissemination.

## Results

### Included studies

**Figure 1:**
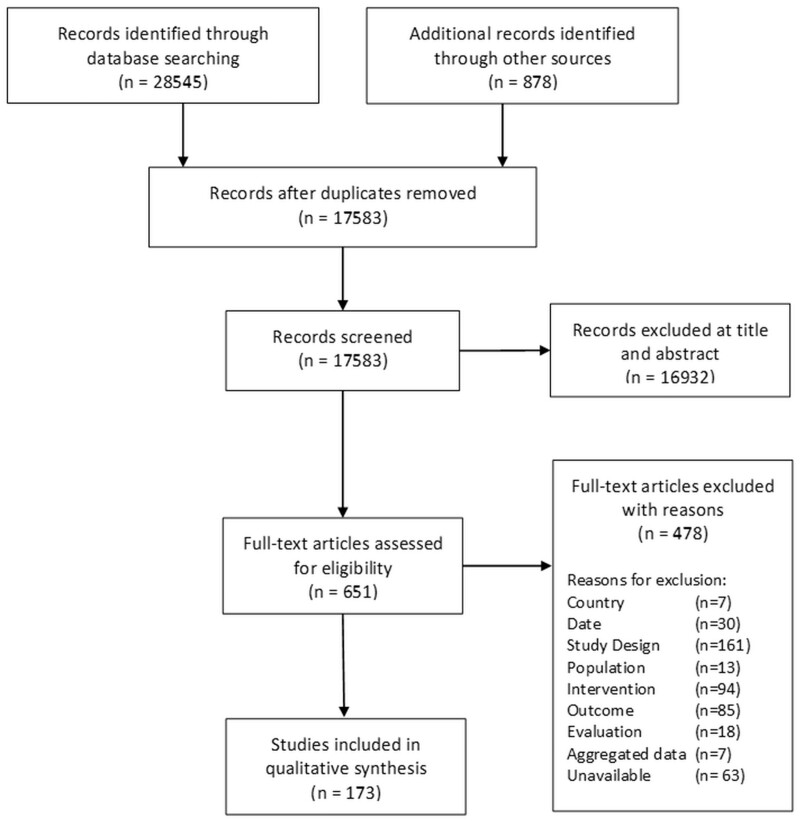
PRISMA—Flow of studies through the scoping review.

A total of 17,578 individual studies were identified and titles and abstracts screened. Of these, 645 were included and screened at full text, resulting in 173 included studies (see Supplementary File S3 for full list), from which data were extracted (see Figure 1 for the flow of studies through the scoping review).

All studies needed to report effect (E** **=** **173) to be included. Most effect data were descriptive (161), some presented effect sizes (70), and five studies measured proximal outcomes, such as number of care plans, that were considered related enough to care entry. Most studies also provided at least some evidence about how an intervention works (Mechanism/Moderator (MM) = 161), describing mechanisms through which it is thought to affect change and the contexts that moderate their effect, in the form of narrative descriptions (164), mediator/moderator analysis (67) or logic models (8). Ninety-three studies described implementation issues (I), including barriers/facilitators (seventy) and activities (forty-nine). Thirty-seven described economic considerations (Ec), mostly some form of cost–benefit analysis. The pattern was the same across both outcomes.

The thematic analysis of intervention type resulted in a categorisation of interventions according to the system-level mechanism—that is the response triggered in participant by the intervention resource in certain contexts to generate the system-level outcome of interest. Thus, interventions were grouped according to the key intervention resource (e.g. shared decision-making meetings) and how it was intended to interact with the reasoning (e.g. thinking, feeling, emotional responses) of people in the system (e.g. shared understanding of child protection concerns and how family strengths can be optimised to address them) to reduce the number of children in care.

The results provide an overview of the total number of studies grouped according to the system-level mechanism, socio-ecological domain and outcome. Some interventions are presented multiple times as they address more than one outcome or have more than one system-level mechanism.

### System-level mechanisms to reduce care

There were nine emergent system-level mechanisms across studies. These were: family or child education/skills building, service integration and/or coordination, new therapeutic approach, practice change (how or what a worker does), structure change (i.e. change to the child welfare system, such as the addition of a new type of court), shared decision-making meetings, mentoring, increase or decrease in a family’s budget and supervision of social workers. The ‘other’ category included studies with system-level mechanisms dissimilar to others (see Supplementary File S4 for full descriptions and included named interventions). While some approaches overlap, and are not fully distinct from each other—for example, offering meetings in a different way can be seen as a therapeutic approach to conducting a group conversation—each category aims to highlight the main system-level mechanism through which the intervention aimed to effect the outcome. Further synthesis of data in each of these categorises in the future suite of research studies informed by this scoping review will lead to a more nuanced and refined conceptualisation of each.

**Figure 2:**
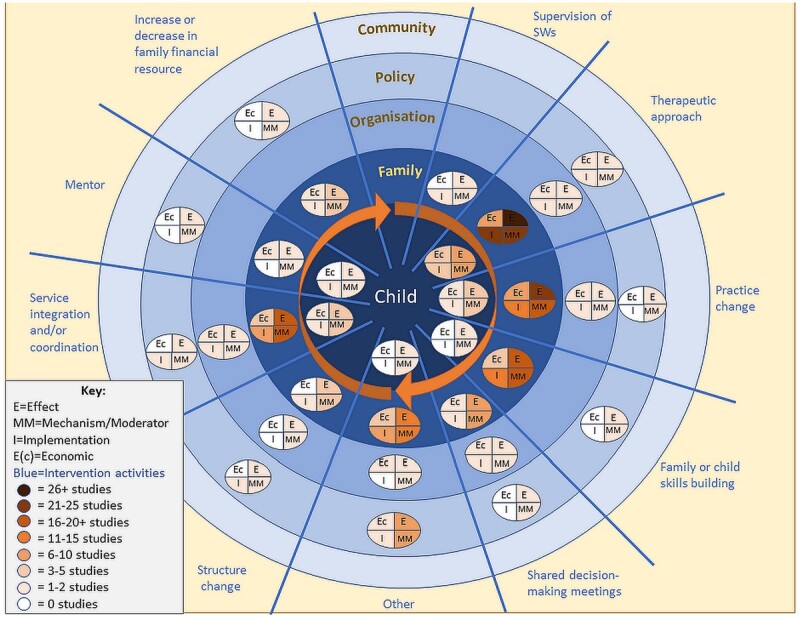
System-level mechanism/socio-ecological domain to reduce care entry and clusters and gaps in their evidence base for EMMIE categories.

**Figure 3:**
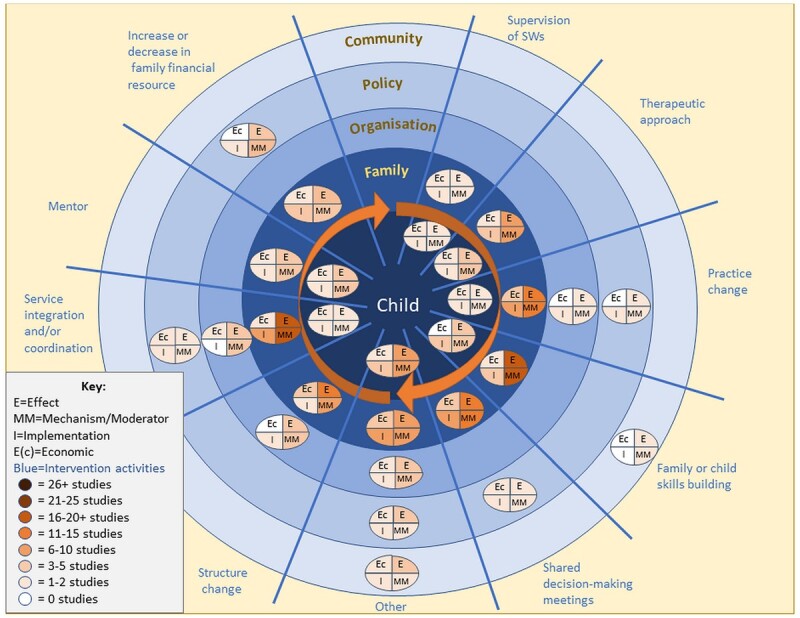
System-level mechanism/socio-ecological domain to increase reunification from care and the gaps and clusters in their evidence base for EMMIE categories.

Evidence maps (Figures 2 and 3) illustrate for each of the system-level mechanisms identified: the socio-ecological domain on which it primarily operates (community, policy, organisation, family or child), the intended outcome (care entry, reunification) and the five EMMIE domains—whether there is evidence in relation to effect (E), how the system-level mechanism works, for whom and under which circumstances (MM), the barriers and facilitators to implementation (I) and economic impact (Ec). For the group of studies that were coded to the ‘other’ category there is no narrative summary because the interventions were not able to be meaningfully grouped in the thematic analysis of intervention type.

#### Summary: reduction in care entry

Ninety-nine studies were found that related to interventions to reduce care entry. The diagram illustrates the main clusters and gaps in the five domains of EMMIE evidence across outcome, system-level mechanism and socio-ecological domain.

There was a cluster of evidence around system-level mechanisms at family level to reduce care entry (Figure 2), with studies examining interventions that worked with the family to reduce the entry of children to care (*n* = 68 studies). There was a smaller cluster of evidence around system-level mechanisms at the level of the child to reduce care entry (*n* = 10 studies). Interestingly, there was only one study aimed at reducing care entry with evidence related to system-level mechanisms at the level of the community.

All types of evidence (EMMIE) were limited in relation to organisational-level mechanisms to reduce care entry (*n* = 5 studies), and policy-level mechanisms to reduce care entry (*n* = 10 studies).

The first cluster of evidence for reducing care entry was around introducing a new therapeutic approach (*n* = 30 studies). Evidence as to whether and how and for whom this system-level mechanism works was most frequently identified at the family level (effect = twenty-six studies; how it works (MM) = twenty-five studies; implementation = twenty-one studies; economic considerations = seven studies) and child level (effect = seven studies; how it works (MM) = seven studies; implementation = five studies; economic considerations = four studies).

The second cluster of evidence was around practitioners changing the way they work (*n* = 25 studies). EMMIE evidence was most frequently identified for this mechanism at the level of family (effect = twenty-one studies; how it works (MM) = twenty studies; implementation = thirteen studies; economic considerations = six studies) and child (effect = four studies; how it works (MM) = two studies; implementation = one study; economic considerations = one study).

A smaller cluster of studies was identified relating to education and/or skills building (*n* = 25 studies). Evidence identified for education and/or skills building aimed at reducing care entry was primarily at the family level (effect = twenty studies, how it works (MM) = twenty studies, implementation = eleven studies, economic considerations = five studies).

Another cluster of evidence examined service integration or coordination around individual needs (*n* = 28 studies). This evidence was mainly focused on coordination around the needs of the family (effect = seventeen studies, how it works (MM) = sixteen studies, implementation = eight studies, economic considerations = five studies) with evidence from three studies focusing on service integration around the needs of the child (effect = three studies, how they worked (MM) = two studies, implementation = one study, economic considerations = one study).

Smaller clusters were found around the system-level mechanism of meetings between social workers and other professionals that include the family in decision making (*n* = 8 studies). Evidence identified in relation to this system-level mechanism related mostly to whether and how it worked at the family level (effect = six studies, how it works (MM) = six studies, implementation = one study, economic considerations = one study). It is notable that there was no evidence found about whether and how this system-level mechanism might work at the level of the child or community to reduce care entry.

Limited evidence was found regarding whether and in what way care entry numbers can be reduced through changes to a family’s financial situation (effect = four studies, how it works (MM) = four studies, implementation = two studies, economic considerations = one study). Of these, three studies provided evidence about how this mechanism operates at the family level, and one at the policy level.

Evidence regarding whether and in what way system-level mechanisms operate at the organisation and policy levels to reduce care entry was more limited (see Figure 2).

Twenty-three studies had too dissimilar system-level mechanisms from others and were grouped as ‘other intervention type’.

#### Summary: reunification from care

Mapping of the data (Figure 3) highlights where evidence clustered by the system-level mechanism/socio-ecological domain/type of evidence (EMMIE).

The spread of evidence about system-level mechanisms to improve reunification from care (Figure 3) is similar to that for care entry, with the biggest clusters around working with the parents/family (*n* = 64 studies) or with the child themselves (*n* = 11 studies). There was less evidence about system-level mechanisms and whether and how they impact on reunification from care at the organisational level (*n* = 9 studies), policy level (*n* = 8 studies) and community level (*n* = 1 study).

The main cluster of evidence for reunification was for the group of interventions that lever the system-level mechanism of education/skill building to support reunification (*n* = 21 studies). Almost exclusively with the education and skill building happening at the family level (effect = eighteen studies; how it works (MM) = seventeen studies; implementation = five studies; economic considerations = zero studies).

A second cluster was evidence related to integration/coordination around parents’/family’s needs (effect = seventeen studies; how it works (MM) = seventeen studies; implementation = eight studies; economic considerations = two studies). Evidence related to whether and how coordination of services around the child’s needs affects reunification was identified in only one study.

Evidence clustered around whether and in what way changes to what a practitioner does with the parents/family can improve reunification from care (effect = fifteen studies; how it works (MM) = fourteen studies; implementation = nine studies; economic considerations = three studies). Again, only one study looked at a change in the way that practitioners work with children/young people.

There were also evidence clusters around family plus practice meetings (*n* = 11 studies), mainly at the family level (effect = eleven studies; how it works (MM) = eleven studies; implementation = eight studies; economic considerations = five studies), structure change, again at the family level (effect = eleven studies; how it works (MM) = ten studies; implementation = two studies; economic considerations = three studies) and new therapeutic approaches (*n* = fourteen studies) mainly with parent(s) (effect = ten studies, how it works (MM) = nine studies, implementation = three studies, economic considerations = one study).

There was an interesting cluster of policy-level EMMIE evidence regarding changes to the financial situation of families (from three studies).

Seventeen studies had too dissimilar system-level mechanisms from others and were grouped as ‘other intervention type’.

## Discussion

This realist scoping review took a more inclusive approach to identifying evidence than traditional approaches, which would exclude studies on the basis of narrow methodological criteria. This resulted in the identification of a large evidence base related to system-level mechanisms that can reduce the number of children in care. While specific named interventions had limited effectiveness data, this systematic scoping review identified a diversity of rich data by grouping interventions by the system-level mechanism that they work through. The originality of using of system-level mechanisms to identify gaps and clusters of evidence is particularly useful in leading to further realist research, which may be helpful in generating knowledge about ‘what works’ to the specific social work context to which it is applied (Rutter and Fisher, 2013).

The evidence map presents clusters and gaps in evidence for whether and in what way system-level mechanisms identified in the thematic analysis reduce the number of children in care. There are important considerations about how areas of research are identified and demarcated. Areas that are theoretically, empirically and practically useful may be located in other bodies of research, particularly in the field of children’s social care which touches on a range of other disciplinary areas. The approach taken in this review to identify system-level mechanisms of interventions is one way of grouping interventions which is intended as a useful departure point for further research. Breadth in scoping reviews is essential, and particularly where system-level mechanisms are under investigation, which will cut across many diverse fields. A challenge essential to any realist review is to bound the potentially limitless scope. Limitations on scope are always required to fit within resource constraints of any research project. It is likely that research from fields beyond the scope of this current review could have changed the spread of identified evidence and thus the gaps and clusters highlighted.

For reducing care entry, the main interventions focused on the relationship between the parent and a practitioner. This either involved a therapeutic approach to working with the parent (delivered by a therapist, or a social work practitioner using the principles/skills of a therapeutic approach they have training in), or a practice change in the way that the social work practitioner interacts with the parent(s). This indicates that the current most commonly evaluated interventions are aimed at creating a behaviour change in a parent using a relationship with a practitioner, or specific skills and approaches.

Other system-level mechanisms were more prevalent for reunification from care. This indicates that possibly achieving this outcome requires different approaches to reducing care entry, and perhaps that the risks are different for children returning to live with their parents. The main system-level mechanism for this outcome was family and/or child education and skill building. Many of the named interventions in this group involved developing parenting skills, so again a focus on parental behaviour change. The second largest system-level mechanism aimed at this outcome was service integration/coordination around the needs of the family. This perhaps shows a recognition of the need for additional services around a family when reintroducing a child to the home, or that additional service involvement may be a prerequisite for reunification.

EMMIE evidence as to what works and in what way to reduce the number of children in care mostly clusters around the operation of system-level mechanisms at the socio-ecological level of the family and the child. This perhaps indicates an individualised approach to child protection. There was a gap in the evidence about which system-level mechanisms reduce the number of children in care and in what way at the community-, organisational- or policy level. This may mean there is a relative lack of outcome evaluations of systemic approaches and that evaluation activity is out of kilter with a more social model of child welfare (Featherstone *et al.*, 2018) that would be more in keeping with traditional social work knowledge and values. The dominance of individualised approaches in evaluations seems to assume that children come into care only because of parental problems, whereas in fact the operation of the child welfare system is a crucial part of the picture, as can be seen by the rising out-of-home care rates in the UK in recent decades. More interventions aimed at that system are therefore needed, along with robust evaluation evidence.

As studies were grouped by system-level mechanism, clusters of evidence show the effect of that *mechanism* (across interventions) on reducing care, rather than the effect of each intervention on reducing care. Of 173 studies, only 70 reported an effect size. This goes some way towards justifying the assumption that study designs in children’s social care research are seldom comparative in nature. Understanding what works about social care practice to better provide social justice and support the human right of families and children to remain together is critical for practice and policy to spend limited resource in ways that are more likely to have positive outcomes for families.

Nearly all studies (*n* = 161) provided some evidence about how the system-level mechanism works, by describing mechanisms through which it is thought to affect change and the contexts that moderate their effect. This indicates there is rich data about theory within the literature. Ninety-three studies described issues around implementation, mainly as barriers and enablers of implementation. The least reported type of evidence was economic considerations (*n* = 37 studies). The EMMIE approach therefore highlighted a lack of economic evaluations across children’s social care research. This could make it difficult for policy makers to make decisions about funding based on research evidence.

As with any scoping review, the review had limitations. One main limitation involves the outcomes of interest. While the intended focus on reduction in children in care was on the safe reduction, rather than simply change in numbers, in fact studies generally included only numbers, so it was not possible in most cases to consider whether interventions were safe. However, by exploring further how these reductions in numbers were achieved, it is hoped that this review helps to conceptualise ‘safe’ reduction. Another limitation is that no attempt was made to assess the quality of studies. There is variability in the quality of studies identified and included grey literature has not been subjected to quality appraisal through peer review process. However, to provide a comprehensive map of the evidence base, it is important to look beyond academic journals. While quality appraisal would be beyond the aims and objectives of a scoping review, a summary of research designs would be helpful to further understand the available literature. It is hoped that a description of evidence types made through the EMMIE framework gives some insight into the types of studies available. While the EMMIE framework was used to shape which evidence was extracted, it was not feasible to synthesise all types of data identified. Further thematic reviews further qualitatively synthesising the mechanism and moderator data are ongoing (see Stabler *et al.*, 2019).

While this approach to a scoping review is novel, and hopefully useful within this field, it is also complicated. The operationalisation of extracting, synthesising and presenting data in a meaningful way necessitated a blending of software packages (NVivo, Microsoft Excel, Microsoft PowerPoint), with regular discussion and reflection on the best approach. As this is the first time the EMMIE framework has been used in a scoping review, there was no practical guide that could be followed. However, it is hoped that this review meets its aim of identifying and grouping approaches taken to reducing the number of children in care in a way that is comprehensive, comprehendible, and useful.

## Conclusion

The gaps in the evidence indicate a need for development, implementation and evaluations of interventions working at the level of the community and policy to reduce the number of children in care. While practice experience tells us that there are many systemic interventions out there, the evidence base indicates that there are few outcome evaluations. More generally there is a need for more comparative study designs where novel interventions are compared with usual services.

This review highlighted clusters of evidence that could be included in systematic reviews. Some have been addressed through the same programme of work (e.g. systematic reviews of the impact on children being in care of intensive family preservation services and shared decision-making meetings (Bezeczky *et al.*, 2020; Nurmatov *et al.*, 2020)). A further question that could be addressed in a future systematic review is, as one of the main intervention types for trying to reduce the need for care, ‘Are parental skill building programmes effective at reducing care entry/improving reunification?’

Descriptions of intervention theory (how it is expected to work to achieve its aims) were generally limited. Better theorising and articulation of this underlying theory in the literature would help future implementers, evaluators and reviewers to understand the evidence base and make decisions based on it. Sharland and Taylor (2006) have made a case for a realist approach to reviewing evidence in social care but very few reviews on children’s social care have followed this approach to date. The greater breadth of a realist approach, with attention to who interventions work for, how and why, has much to offer researchers and practitioners, compared to more traditional approaches to systematic review based on effect only.

## Supplementary Material

bcab213_Supplementary_Data
