# Supplementary material for: A scoping review of system-level mechanisms to prevent children being in out-of-home care
Source: Br J Soc Work. 2021 Nov 9;52(5):2515–36. doi: 10.1093/bjsw/bcab213 (PMC9847665; doi:10.1093/bjsw/bcab213)
Supplement: bcab213_Supplementary_Data [file bcab213_supplementary_data.zip › Supplementary file 1.docx]

| PICO (E) | Inclusion criteria |
| --- | --- |
| Population | Children and young people in need of care or who have been in care when ≤18 years old. |
| Intervention | Interventions are defined as a disruption to the system. They can operate across a single or multiple socio-ecological domain/s: intra-personal; inter-personal; organisational; community; and policy. Interventions could be described in terms of their theory of change, activities and/or resources (e.g. they range from a discrete package with pre-specified resources and activities to a more generic, contextually flexible theory of change). |
| Comparator | Usual care; alternative intervention; no comparator |
| Outcome | - Number of children and young people entering care - Number of children and young people (re-)entering care - Number of children and young people re-unified with their families following a period in statutory care   Corollary or proximal outcomes that support these outcome measures. |
| Evaluation | Evaluation of the intervention is reported for one or more EMMIE dimensions:   - Effectiveness (E) - Mechanisms through which the intervention generates intended or unintended effects (M) - Contexts that moderate effects (M) - System determinants of implementation (I) - Economic effectiveness (E) |

Table 1: Table 1: PICO (E) table illustrating scoping review eligibility criteria
