# Supplementary material for: A scoping review of system-level mechanisms to prevent children being in out-of-home care
Source: Br J Soc Work. 2021 Nov 9;52(5):2515–36. doi: 10.1093/bjsw/bcab213 (PMC9847665; doi:10.1093/bjsw/bcab213)
Supplement: bcab213_Supplementary_Data [file bcab213_supplementary_data.zip › Supplementary file 2.docx]

Supplementary File 2: Search terms and databases

Websites searched:

Action for Children, Barnardo’s, Care Leavers’ Association, Children’s Commissioners’ offices for four UK nations, Children’s Society, Child Welfare Information Gateway, Department for Education, Early Intervention Foundation, Joseph Rowntree Foundation, National Institute for Health and Care Excellence (NICE), OpenGrey, REES Centre, Samaritans, Thomas Coram Foundation.

Databases searched:

| Who | Database | Dates coverage dates (if provided) | Results |
| --- | --- | --- | --- |
| Ovid Platform | | | |
| FM | Medline | 1946 to March 27, 2018 | 4042 |
| FM | Medline in Process | March 27, 2018 | 344 |
| FM | Medline ePub | March 27, 2018 | 124 |
| FM | Social Policy & Practice | 201801 | 1275 |
| FM | PsycINFO | 1806 to March Week 3 2018 | 2894 |
| LS | Embase | 1947 to present | 4353 |
| LS | HMIC | No date provided | 8 |
| EBSCO Platform | | | |
| LS/FM | CINAHL | No date provided | 1512 |
| FM | ERIC | No date provided | 1325 |
| FM | BEI | Database start = 2011 | 60 |
| FM | Child Development and Adolescent Studies | No date provided | 1463 |
| ProQuest Platform (5 April 2018) | | | |
| ALW/FM | ASSIA |  | 892 |
| ALW/FM | IBSS |  | 1052 |
| ALW/FM | Sociological Abstracts (includes Social Services Abstracts) |  | 1138 |
|  |  |  |  |
| LS/FM | Scopus |  | 1821 |
| Web of Science (ISI Common format for inport) | | | |
| FM |  |  | 6242 |

Total = 28545

Search terms used:

**OVID Platform (Medline – adapted to Social Policy & Practice, PsycINFO, Embase, HMIC)**

| 1. | (teen or teens or teenage*).tw |
| --- | --- |
| 2 | (adolesc* or preadolesc* or pre-adolesc* or juvenil*).tw. |
| 3 | (youth or youths or youngster*).tw. |
| 4 | ((young adj (person* or persons or people)) or early adult*).tw. |
| 5 | (student or students or schoolchild*).tw. |
| 6 | exp infant/ |
| 7 | exp Child/ |
| 8 | Young adult/ |
| 9 | adolescent/ |
| 10 | (boy* or girl* or child or children or infant or infants or kid or kids).tw. |
| 11 | (pediatri* or paediatri*).tw. |
| 12 | (pubescen* or puberty).tw. |
| 13 | orphan*.tw. |
| 14 | (adopt* adj5 (child or children)).tw. |
| 15 | Child, Orphaned/ |
| 16 | Child of impaired parents/ |
| 17 | or/1-16 |
| 18 | ((substitute or local authority or out of home or public or order or place* or group) adj (care or placement*)).tw. |
| 19 | ((nonparent or non-parent) adj3 care).tw. |
| 20 | ((institution* or residential or foster or kinship or group) adj3 (care or home* or placement)).tw. |
| 21 | ((children's or childrens) adj home).tw. |
| 22 | support* living.tw. |
| 23 | looked after.tw. |
| 24 | Special guardian*.tw. |
| 25 | In care.tw. |
| 26 | Edge of care.tw. |
| 27 | welfare care.tw. |
| 28 | or/18-27 |
| 29 | 17 and 28 |
| 30 | ((family preservation or preserv* famil* or multi systemic) adj5 (intensive or team or service* or project* or program*or therap*)).tw. |
| 31 | (family recovery adj5 (intensive or team or service* or project* or program*or therap*)).tw. |
| 32 | familyadj5 reunif*.tw. |
| 33 | ((family support* or support*famil*) adj5 (intensive or team or service* or project* or program*or therap*)).tw. |
| 34 | (prevent* adj3 (placement* or placing)).tw. |
| 35 | (care adj5 placement prevent*).tw. |
| 36 | (Family adj5 (home-based or in-home)).tw. |
| 37 | ((family or home) adj (visiting or visitation)).tw. |
| 38 | Homebuilders.tw. |
| 39 | Famil* First.tw. |
| 40 | Team around the family.tw. |
| 41 | ((family nurse or nurse family) adj partner*).tw. |
| 42 | (family intervention adj5 (intensive or team or service* or project* or program*or therap*)).tw. |
| 43 | (family crisis intervention adj5 (intensive or team or service* or project* or program*or therap*)).tw. |
| 44 | Signs of Safety.tw. |
| 45 | (Reclaiming Social Work or Hackney model).tw. |
| 46 | Minding the Baby.tw. |
| 47 | (Florida and (Infant or Young Child) and Mental Health and (Pilot or Plan)).tw. |
| 48 | Video Interaction Guidance.tw. |
| 49 | "Family Drug and Alcohol Court".tw. |
| 50 | Parent* Under Pressure.tw. |
| 51 | Parenting Plus.tw. |
| 52 | (Targeted support or Targeted youth support).tw. |
| 53 | (Family Reunification adj5 (intensive or team or service* or project* or program*or therap*)).tw. |
| 54 | family partnership model*.tw. |
| 55 | family group conferenc*.tw. |
| 56 | family group decision-making.tw. |
| 57 | (Parent Child adj (atunment or Interaction Therap*)).tw. |
| 58 | (Solution* Focussed Brief Therap* or Solution* Focused Brief Therap*).tw. |
| 59 | ((family focused or family focussed or multi-systemic or multisystemic) adj (therap* or casework)).tw. |
| 60 | Parent Infant Psychotherapy.tw. |
| 61 | (pause adj5 (team or service* or project* or program*or therap*)).tw. |
| 62 | ((family preservation or preserv* famil*) adj5 (intensive or team or service* or project* or program*or therap*)).tw. |
| 63 | Family relations/ |
| 64 | Parent-Child relations/ |
| 65 | Child Protective Services/ |
| 66 | (child adj (protection or protective)).tw. |
| 67 | or/30-66 |
| 68 | (((Case-control or cohort or longitudinal or comparative or controlled or cross-sectional or pilot or evaluation or feasibility or follow-up) adj (trial* or study or studies)) or Interrupted time series).tw. |
| 69 | (randomized controlled trial or controlled clinical trial).pt. or (randomized or randomised).ab. or placebo.ab. or randomly.ab. or trial.ti. |
| 70 | (systematic adj (review or reviews)).tw. |
| 71 | (meta-analysis or metaanalysis).tw. |
| 72 | or/68-71 |
| 73 | 67 or 72 |
| 74 | 29 and 73 |
| 75 | exp animals/ not humans.sh. |
| 76 | 74 not 75 |
| 77 | limit 76 to yr="1991 -Current" |

**EBSCO PLATFORM (CINAHL, ERIC, BEI, Child Development and Adolescent Studies)**

| **#** | **Query** |
| --- | --- |
| S74 | S28 AND S72 Limiters - Published Date: 19910101-20180331; Human |
| S72 | S66 OR S71 |
| S71 | S68 OR S69 OR S70 OR S71 |
| S70 | AB (meta-analysis or metaanalysis) |
| S69 | AB (systematic N (review or reviews)) |
| S68 | PT (("randomized controlled trial" or "controlled clinical trial")) OR AB ((randomized or randomised)) OR AB placebo OR AB randomly OR TI trial |
| S67 | AB (((“Case-control” or cohort or longitudinal or comparative or controlled or cross-sectional or pilot or evaluation or feasibility or follow-up) N (trial* or study or studies)) or “Interrupted time series”) |
| S66 | S29 OR S30 OR S31 OR S32 OR S33 OR S34 OR S35 OR S36 OR S37 OR S38 OR S39 OR S40 OR S41 OR S42 OR S43 OR S44 OR S45 OR S46 OR S47 OR S48 OR S49 OR S50 OR S51 OR S52 OR S53 OR S54 OR S55 OR S56 OR S57 OR S58 OR S59 OR S60 OR S61 OR S62 OR S63 OR S64 OR S65 |
| S65 | AB (child N (protection or protective)) |
| S64 | AB "Child Protective Services" |
| S63 | (MH "Parent-Child Relations") |
| S62 | (MH "Family Relations") |
| S61 | AB ((family preservation or preserv* famil*) N5 (intensive or team or service* or project* or program*or therap*)) |
| S60 | AB (pause N5 (team or service* or project* or program*or therap*)) |
| S59 | AB "Parent Infant Psychotherapy |
| S58 | AB ((family focused or family focussed or multi-systemic or multisystemic) N (therap* or casework)) |
| S57 | AB (Solution* Focussed Brief Therap* or Solution* Focused Brief Therap*) |
| S56 | TX (Parent Child N (attunement or Interaction Therap*)) |
| S55 | AB family group decision-making |
| S54 | AB family group conferenc* |
| S53 | AB family partnership model* |
| S52 | AB (Family Reunification N5 (intensive or team or service* or project* or program*or therap*)) |
| S51 | AB ("Targeted support" or "Targeted youth support") |
| S50 | AB "Parenting Plus" |
| S49 | AB "Parent* Under Pressure" |
| S48 | AB "Family Drug and Alcohol Court" |
| S47 | AB "Video Interaction Guidance" |
| S46 | AB (Florida and (Infant or Young Child) and "Mental Health" and (Pilot or Plan)) |
| S45 | AB "Minding the Baby" |
| S44 | AB ("Reclaiming Social Work" or "Hackney model") |
| S43 | AB "Signs of Safety" |
| S42 | AB (family crisis intervention N5 (intensive or team or service* or project* or program*or therap*)) |
| S41 | AB ("family intervention" N5 (intensive or team or service* or project* or program*or therap*)) |
| S40 | AB ((family nurse or nurse family) N partner*) |
| S39 | AB "Team around the family" |
| S38 | AB "Famil* First" |
| S37 | AB Homebuilders |
| S36 | AB ((family or home) N (visiting or visitation)) |
| S35 | AB (Family N5 (home-based or in-home)) |
| S34 | AB (care N5 placement prevent*) |
| S33 | AB (prevent* N3 (placement* or placing)) |
| S32 | AB (("family support*" or "support* famil*") N5 (intensive or team or service* or project* or program*or therap*)) |
| S31 | AB family N5 reunif* |
| S30 | AB ("family recovery" N5 (intensive or team or service* or project* or program*or therap*)) |
| S29 | AB ((family preservation or preserv* famil* or multi systemic) N5 (intensive or team or service* or project* or program*or therap*)) |
| S28 | S17 AND S27 |
| S27 | S18 OR S19 OR S20 OR S21 OR S22 OR S23 OR S24 OR S25 OR S26 |
| S26 | AB "welfare care" |
| S25 | AB "Edge of care" |
| S24 | AB "Special guardian*" |
| S23 | AB "looked after" |
| S22 | AB "support* living" |
| S21 | AB ((children's or childrens) N home) |
| S20 | AB ((institution* or residential or foster or kinship or group) N3 (care or home* or placement)) |
| S19 | AB ((nonparent or non-parent) N3 care) |
| S18 | AB ((substitute or local authority or out of home or public or order or place* or group) N (care or placement*)) |
| S17 | S1 OR S2 OR S3 OR S4 OR S5 OR S6 OR S7 OR S8 OR S9 OR S10 OR S11 OR S12 OR S13 OR S14 OR S15 OR S16 |
| S16 | (MH "Children of Impaired Parents") |
| S15 | (MH "Orphans and Orphanages") |
| S14 | AB (adopt* N5 (child or children)) |
| S13 | AB orphan* |
| S12 | AB (pubescen* or puberty) |
| S11 | AB (pediatri* or paediatri*) |
| S10 | AB (boy* or girl* or child or children or infant or infants or kid or kids) |
| S9 | (MH "Adolescence") |
| S8 | (MH "Young Adult") |
| S7 | (MH "Child+") |
| S6 | (MH "Infant+") |
| S5 | AB (student or students or schoolchild*) |
| S4 | AB ((young N (person* or persons or people)) or early adult*) |
| S3 | AB (youth or youths or youngster*) |
| S2 | AB (adolesc* or preadolesc* or pre-adolesc* or juvenil*) |
| S1 | AB (teen or teens or teenage*) |

**Proquest Databases (ASSIA, IBIS, Sociological Abstracts)**

| ab(teen OR teens OR teenage* OR adolesc* OR preadolesc* OR pre-adolesc* OR juvenil* OR youth OR youths OR youngster* OR "young person*" OR "young persons" OR "young people" OR "early adult*" OR student OR students OR schoolchild* OR boy* OR girl* OR child OR children OR infant OR infants OR kid OR kids OR pediatri* OR paediatri* OR pubescen* OR puberty OR orphan*) OR ab((adopted NEAR/5 child) OR (adopted NEAR/5 children)) |
| --- |
| ab((substitute OR "local authority" OR "out of home" OR public OR order OR place* OR group) NEAR/2 (care OR placement*)) OR ab((nonparent OR non-parent) NEAR/3 care) OR ab((institution* OR residential OR foster OR kinship OR group) NEAR/3 (care OR home* OR placement)) OR ab((children's OR childrens) NEAR/1 home) OR ab("support* living" OR "looked after" OR "special guardian*" OR "in care" OR "edge of care" OR "welfare care") |
| 1 AND 2 |
| ab("family intervention" NEAR/5 (intensive OR team OR service* OR project* OR program* OR therap*)) OR ab("family crisis intervention" NEAR/5 (intensive OR team OR service* OR project* OR program* OR therap*)) OR ab("signs of safety" OR "reclaiming social work" OR "hackney model" OR "minding the baby") OR ab((Florida AND (Infant OR "Young Child") AND "Mental Health" AND (Pilot OR Plan))) OR ab("Video Interaction Guidance" OR "Family Drug and Alcohol Court" OR "Parent* Under Pressure" OR "Parenting Plus" OR "Targeted support" OR "Targeted youth support") OR ab("Family Reunification" NEAR/5 (intensive OR team OR service* OR project* OR program* OR therap*)) OR ab("family partnership model*" OR "family group conferenc*" OR "family group decision-making") OR ab("Parent Child" NEAR/1 (atunement OR "Interaction Therap*")) OR ab("Solution* Focussed Brief Therap*") OR ab(("family focused" OR "family focussed" OR "multi-systemic" OR multisystemic) NEAR/1 (therap* OR casework)) |
| (ab(("family preservation" OR "preserv* famil*" OR "multi systemic") NEAR/5 (intensive OR team OR service* OR project* OR program* OR therap*)) OR ab("family recovery" NEAR/5 (intensive OR team OR service* OR project* OR program* OR therap*)) OR ab(family NEAR/5 reunif*) OR ab(("family support*" OR "support*famil*") NEAR/5 (intensive OR team OR service* OR project* OR program* OR therap*)) OR ab(prevent* NEAR/3 (placement* OR placing)) OR ab(care NEAR/5 "placement prevent*") OR ab(Family NEAR/5 ("home-based" OR "in-home")) OR ab((family OR home) NEAR/1 (visiting OR visitation)) OR ab(homebuilders OR "famil* first" OR "team around the family") OR ab(("family nurse" OR "nurse family") NEAR/1 partner*)) |
| ab("Parent Infant Psychotherapy") OR ab(pause NEAR/5 (team OR service* OR project* OR program* OR therap*)) OR ab(("family preservation" OR "preserv* famil*") NEAR/5 (intensive OR team OR service* OR project* OR program* OR therap*)) OR ab(child NEAR/1 (protection OR protective)) |
| ab("Interrupted time series" or "randomized controlled trial" or "controlled clinical trial" or randomized or randomised or placebo or randomly ) OR ti(trial) OR ab((systematic N/1 (review or reviews)).) OR ab("meta analysis" or metaanalysis) |
| 4 OR 5 OR 6 OR 7 |
| 3 AND 8 |
| Limit to Scholarly journals and 1990-2019 |

**Scopus**

1. (teen or teens or teenage*) or (adolesc* or preadolesc* or pre-adolesc* or juvenil*) or (youth or youths or youngster*) - 500,515
2. Young w/1 (person* or persons or people) or “early adult*” or (boy* or girl* or child or children or infant or infants or kid or kids) or (pediatri* or paediatri*) or (pubescen* or puberty) or orphan* or (student or students or schoolchild*) – 167,985
3. #1 or #2 – 638,154
4. ((substitute or “local authority” or “out of home” or public or order or place* or group) w/1 (care or placement*)) or ((nonparent or non-parent) w/3 care) or ((institution* or residential or foster or kinship or group) w/3 (care or home* or placement)) – 275,773
5. “support* living” or “looked after” or “Special guardian” or “in care” or “edge of care” or “welfare care” – 14,792
6. ((children's or childrens) w/1 home) - 5,220
7. #4 or #5 or #6 – 292,957
8. #3 and #7 – 10,809
9. ((“family preservation” or “preserv* famil*” or “multi systemic”) w/5 (intensive or team or service* or project* or program* or therap*)) or (“family recovery” w/5 (intensive or team or service* or project* or program* or therap*)) or (family w/5 reunif*) SPLIT HERE 958
10. ((“family support*” or “support*famil*”) w/5 (intensive or team or service* or project* or program* or therap*)) – 951
11. (prevent* w/3 (placement* or placing)) or (care w/5 “placement prevent*”) or (Family w/5 (home-based or in-home)) or ((family or home) w/1 (visiting or visitation)) or Homebuilders or “Famil* First” or “Team around the family” 4786
12. ((("family nurse" OR "nurse family") W/1 partner*) OR (("family intervention" OR "family crisis intervention") W/5 (intensive OR team OR service* OR project* OR program* OR therap*))) 541
13. “Signs of Safety” or “Reclaiming Social Work” or “Hackney model” or “Minding the Baby” or “Video Interaction Guidance” or "Family Drug and Alcohol Court" or “Parent* Under Pressure” or “Parenting Plus” 84
14. (Florida and (Infant or “Young Child”) and “Mental Health” and (Pilot or Plan)) 1
15. “Targeted support” or “Targeted youth support” or (“Family Reunification” w/5 (intensive or team or service* or project* or program* or therap*)) 353
16. “family partnership model*” or “family group conferenc*” or “family group decision-making” or (“Parent Child” w/1 (attunement or “Interaction Therap*”)) or “Solution* Focussed Brief Therap*” or “Solution* Focused Brief Therap*” 564
17. ((“family focused” or “family focussed” or multi-systemic or multisystemic) w/1 (therap* or casework)) or “Parent Infant Psychotherapy” – 436
18. (pause w/5 (team or service* or project* or program* or therap*)) or ((“family preservation” or “preserv* famil*”) w/5 (intensive or team or service* or project* or program* or therap*)) 401
19. (child w/1 (protection or protective) – 6,654
20. #9 or #10 or #11 or #12 or #13 – 17,988
21. (((Case-control or cohort or longitudinal or comparative or controlled or cross-sectional or pilot or evaluation or feasibility or follow-up) w/1 (trial* or study or studies)) or “Interrupted time series”) or (“randomized controlled trial” or “controlled clinical trial”) or (randomized or randomised) or placebo or randomly or (systematic w/1 (review or reviews)) or (meta-analysis or metaanalysis) – 1,965,841
22. #14 or #15 – 1,978,838
23. #8 and #16 – 1894 (1821 with date restrictions)

**Web of Science**

| # 18 | #15 AND #6  **Refined by:** **DOCUMENT TYPES:** ( ARTICLE OR REVIEW ) AND **WEB OF SCIENCE CATEGORIES:** ( SOCIAL WORK OR FAMILY STUDIES OR PEDIATRICS OR ETHICS OR PSYCHOLOGY DEVELOPMENTAL OR NURSING OR SOCIAL ISSUES OR PSYCHIATRY OR HEALTH POLICY SERVICES OR MEDICAL ETHICS OR REHABILITATION OR EDUCATION EDUCATIONAL RESEARCH OR DEMOGRAPHY OR PUBLIC ADMINISTRATION OR SOCIAL SCIENCES BIOMEDICAL OR SOCIAL SCIENCES INTERDISCIPLINARY OR PSYCHOLOGY OR ETHNIC STUDIES OR SUBSTANCE ABUSE OR SOCIOLOGY OR PRIMARY HEALTH CARE OR BEHAVIORAL SCIENCES OR PSYCHOLOGY APPLIED )  Indexes=SSCI, CPCI-SSH, ESCI Timespan=1991-2018 |
| --- | --- |
| # 17 | #15 AND #6  **Refined by:** **DOCUMENT TYPES:** ( ARTICLE OR REVIEW )  Indexes=SSCI, CPCI-SSH, ESCI Timespan=1991-2018 |
| # 16 | #15 AND #6  Indexes=SSCI, CPCI-SSH, ESCI Timespan=1991-2018 |
| # 15 | #11 OR #10 OR #9 OR #8 OR #7  Indexes=SSCI, CPCI-SSH, ESCI Timespan=1991-2018 |
| # 14 | #13 AND #6  Indexes=SSCI, CPCI-SSH, ESCI Timespan=1991-2018 |
| # 13 | #12 OR #11 OR #10 OR #9 OR #8 OR #7  Indexes=SSCI, CPCI-SSH, ESCI Timespan=1991-2018 |
| # 12 | TS=((Case-control or cohort or longitudinal or comparative or controlled or cross-sectional or pilot or evaluation or feasibility or follow-up) SAME (trial* or study or studies)) or TS=(“Interrupted time series” or “randomized controlled trial” or “controlled clinical trial” or randomized or randomised or placebo or randomly or systematic review* or meta-analysis or metaanalysis)  Indexes=SSCI, CPCI-SSH, ESCI Timespan=1991-2018 |
| # 11 | TS=(pause SAME (team or service* or project* or program* or therap*)) or TS=((“family preservation” or “preserv* famil*”) SAME (intensive or team or service* or project* or program* or therap*)) or TS=("child protection" or “Child Protective Services”)  Indexes=SSCI, CPCI-SSH, ESCI Timespan=1991-2018 |
| # 10 | TS=("Targeted support" or "Targeted youth support") or TS=("Family Reunification" SAME (intensive or team or service* or project* or program* or therap*)) or TS=("family partnership model*" or "family group conferenc*" or "family group decision-making" or "Parent Infant Psychotherapy") or TS=("Parent Child" SAME (attunement or Interaction Therap*)) or TS=("Solution* Focussed Brief Therap*" or "Solution* Focused Brief Therap*") or TS=(("family focused" or "family focussed" or multi-systemic or multisystemic) SAME (therap* or casework))  Indexes=SSCI, CPCI-SSH, ESCI Timespan=1991-2018 |
| # 9 | TS=("Signs of Safety" or "Reclaiming Social Work" or "Hackney model" or "Minding the Baby" or "Video Interaction Guidance" or "Family Drug and Alcohol Court" or "Parent* Under Pressure" or "Parenting Plus") or TS=(Florida AND (Infant or "Young Child") and "Mental Health" and (Pilot or Plan))  Indexes=SSCI, CPCI-SSH, ESCI Timespan=1991-2018 |
| # 8 | TS=(Homebuilders or "Famil* First" or "Team around the family" or "family nurse partner*" or "nurse family partner*") or TS=("family intervention" SAME (intensive or team or service* or project* or program* or therap*)) or tS=("family crisis intervention" SAME (intensive or team or service* or project* or program* or therap*))  Indexes=SSCI, CPCI-SSH, ESCI Timespan=1991-2018 |
| # 7 | TS=((“family preservation” or “preserv* famil*” or “multi systemic”) SAME (intensive or team or service* or project* or program* or therap*)) or TS=(“family recovery” SAME (intensive or team or service* or project* or program* or therap*)) or TS=(family SAME reunif*) or TS=(family support* SAME (intensive or team or service* or project* or program* or therap*)) or TS=(support*famil* SAME (intensive or team or service* or project* or program* or therap*)) or TS=(prevent* SAME (placement* or placing)) or TS=(care SAME placement prevent*) or TS=(Family SAME (home-based or in-home)) or TS=(family SAME (visiting or visitation)) or TS=(home SAME (visiting or visitation))  Indexes=SSCI, CPCI-SSH, ESCI Timespan=1991-2018 |
| # 6 | #5 AND #1  Indexes=SSCI, CPCI-SSH, ESCI Timespan=1991-2018 |
| # 5 | #4 OR #3 OR #2  Indexes=SSCI, CPCI-SSH, ESCI Timespan=1991-2018 |
| # 4 | TS=(children's home or childrens home)  Indexes=SSCI, CPCI-SSH, ESCI Timespan=1991-2018 |
| # 3 | TS=( “support* living” or “looked after” or “Special guardian” or “in care” or “edge of care” or “welfare care”)  Indexes=SSCI, CPCI-SSH, ESCI Timespan=1991-2018 |
| # 2 | TS=((substitute or “local authority” or “out of home” or public or order or place* or group) SAME (care or placement*)) or TS=((nonparent or non-parent) SAME care)  Indexes=SSCI, CPCI-SSH, ESCI Timespan=1991-2018 |
| # 1 | TS=(teen or teens or teenage* or adolesc* or preadolesc* or pre-adolesc* or juvenil*or youth or youths or youngster*or "young person" or "young persons" or "young people" or "early adult*" or boy* or girl or child or children or infants or infant or kid or kids or pediari* or paediatri* or pubescen* or puberty or orphan* or “orphaned child” or “child of impaired parent” or student or students or schoolchild*)  Indexes=SSCI, CPCI-SSH, ESCI Timespan=1991-2018 |
