# Supplementary material for: A scoping review of system-level mechanisms to prevent children being in out-of-home care
Source: Br J Soc Work. 2021 Nov 9;52(5):2515–36. doi: 10.1093/bjsw/bcab213 (PMC9847665; doi:10.1093/bjsw/bcab213)
Supplement: bcab213_Supplementary_Data [file bcab213_supplementary_data.zip › Supplementary_file_4_updated.docx]

Supplementary file 4: Summary of evidence for intervention types

| Intervention type | | | | | |
| --- | --- | --- | --- | --- | --- |
| System-level mechanism | | **Socioeconomic domain across outcome** | | | **Named interventions in group** |
|  |  |  | Care entry | Reun from care |  |
| *Education/ skill building*  *n=44* | System-level mechanism: teaching parents using a variety of approaches can result in the learning of new skills that change whether the child may be cared for safely at home. The most common aim of these programmes was to improve parenting skills. | Child | 2 | 5 | The Strengthening Families Programme, Homebuilders, some Intensive Family Preservation Services, some Reunification Services, Parent Mutual Aid, Shared Parenting program, Functional Family Therapy-Child Welfare. |
|  |  | Family | 20 | 18 |  |
|  |  | Organisation | 0 | 0 |  |
|  |  | Policy | 2 | 0 |  |
|  |  | Community | 0 | 1 |  |
| *Needs-based service integration/ coordination*  *n=43* | System-level mechanism: coordinating services around the needs of a child or family can interact with the reasoning of the child, family, and professionals through a chain of causality and change whether a child will enter care or be reunified with their family. Approaches in this category mainly involved a form of case management. Often part of a wider programme of work with the family. | Child | 3 | 1 | Dependency Drug Courts, Family Drug Courts/ Family Treatment Drug Courts, Family Drug and Alcohol Courts, Multi-Systemic Therapy, multi-systems approaches, Delta Method, some (Intensive/) Family Preservation Services, Differential Response, Intensive Family Support, Transitioning Youth to Families, Intensive Case Management, Intensive Home-Based Services, Family-Centred Out-of-Home Care, and Shared Parenting. |
|  |  | Family | 17 | 17 |  |
|  |  | Organisation | 2 | 3 |  |
|  |  | Policy | 1 | 2 |  |
|  |  | Community | 0 | 0 |  |
| *Therapeutic approach n=42 studies* | System-level mechanism: the use of a therapeutic approach for child or adult carer, underpinned by a therapeutic model, brings about changes in family thinking, feeling, and acting that will impact whether a child needs to enter care or be reunified with their family. These interventions were delivered by specifically trained professionals, rather than as part of more general social work practice. Some included service coordination (i.e. MST) but from a therapeutic base. | Child | 7 | 3 | Multi-Systemic Therapy (MST), MST–Child Abuse and Neglect, Building Stronger Families, Multi-Dimensional Family Therapy, Cognitive-Behavioural Approach, CAT, couple and family therapy, Treatment Foster Care, Child-Parent Psychotherapy, Families First – intensive in-home therapy, Building Blocks Psychodynamic Treatment Approach, Functional Family Therapy (FFT), FFT-Child Welfare. |
|  |  | Family | 26 | 10 |  |
|  |  | Organisation | 0 | 0 |  |
|  |  | Policy | 1 | 0 |  |
|  |  | Community | 1 | 0 |  |
| *Change in what a worker does (practice change)*  *n=42 studies* | System-level mechanism: the worker changing the way that they interact with the family (for example using a strengths-based approach) interacts with how the family think, feel, and act in ways that can changes whether a child will enter care or be reunified with their family. While these approaches may be underpinned by therapeutic theories, interventions in this category did not rely solely on a therapeutic model. | Child | 4 | 2 | Some (Intensive/) Family Preservation Services, some Family Reunification Services, Homebuilders, Triple P Positive Parenting Programme, Promoting First Relationships, Family Partnership Model, Sobriety Treatment and Recovery Teams, Signs of Safety. |
|  |  | Family | 21 | 15 |  |
|  |  | Organisation | 2 | 1 |  |
|  |  | Policy | 2 | 1 |  |
|  |  | Community | 1 | 0 |  |
| *Structure change in the child welfare system n=18 studies* | System-level mechanism: a change in the structure within which child welfare operates interacts with the reasoning of the people in child welfare system to bring about changes in whether a child will enter care or be reunified with their family. The interventions were mostly drug-related court interventions for parents. | Child | 0 | 0 | Dependency Drug Court, Family Drug Court, Family Treatment Drug Court, Dependency Treatment Court, Family Drug and Alcohol Court, and Unified Family Courts. |
|  |  | Family | 3 | 11 |  |
|  |  | Organisation | 1 | 4 |  |
|  |  | Policy | 1 | 0 |  |
|  |  | Community | 0 | 0 |  |
| *Meetings that include the family and relevant workers*  *n=16 studies* | System-level mechanism: a meeting between family networks and workers that includes the family in decision making interacts with the reasoning of the family, child, social worker, and other involved professionals to create a change in whether the family keeps their child at home. | Child | 0 | 0 | Family First, Team Decision Making, Family Group Conferences, Family Group Decision-Making, Family Team Conferencing, Family Involvement Meeting, and Family Group Engagement |
|  |  | Family | 6 | 11 |  |
|  |  | Organisation | 2 | 0 |  |
|  |  | Policy | 1 | 2 |  |
|  |  | Community | 0 | 0 |  |
| *Interventions that increase/*  *decrease a family's finances*  *n=13 studies* | System-level mechanism: changes in the household financial resources interact with a family’s reasoning and result in a change in their feelings and behaviour toward their child/ren and this can affect the need for care. This group included interventions that directly increased or decreased a family's finances, including welfare reforms. | Child | 0 | 0 | Mostly add-on elements of larger interventions such as Family Preservation Services: Emergency Assistance Funds (e.g. Homebuilders), Family Assistance Fund, and some welfare reforms. |
|  |  | Family | 4 | 5 |  |
|  |  | Organisation | 0 | 0 |  |
|  |  | Policy | 2 | 3 |  |
|  |  | Community | 0 | 0 |  |
| *Mentoring*  *n=10 studies* | System-level mechanism: a relationship between a parent and a person who has shared or similar life experience helps the person who used services to change, or engage with the services offered, or to learn new skills and helps them to keep their child at home safely. These interventions were all provided as part of a larger programme of work. | Child | 2 | 3 | Mentors/life coaches/recovery coaches as add-on to larger interventions, such as FDAC or No Wrong Door; and community mentoring interventions, such as foster carer to parent, community member to parent, family to family, previous drug user to parent with drug misuse. |
|  |  | Family | 2 | 5 |  |
|  |  | Organisation | 0 | 0 |  |
|  |  | Policy | 1 | 0 |  |
|  |  | Community | 0 | 0 |  |
| *Supervision of social workers*  *n=3 studies* | System-level mechanism: the way in which supervision of a social worker interacts with their reasoning to change the way that they work with a family can help a family to keep their child safely at home. | Child | 0 | 1 | Supervision as part of the On the Way Home program, Building Blocks, and Functional Family Therapy-Child Welfare. |
|  |  | Family | 2 | 2 |  |
|  |  | Organisation | 0 | 0 |  |
|  |  | Policy | 0 | 0 |  |
|  |  | Community | 0 | 0 |  |
| *Other intervention type n=39 studies* | System-level mechanism: various | Child | 1 | 6 | Respite care / crisis nurseries / pre-school and day care, specialist home visiting / nursing services, Community-Based Alternative Response Systems/ Differential Response, increased court reviews. Performance-based managed care contracting, homework support / school-based educational support, Parent Mutual Aid Organisations. |
|  |  | Family | 14 | 10 |  |
|  |  | Organisation | 1 | 3 |  |
|  |  | Policy | 6 | 4 |  |
|  |  | Community | 0 | 1 |  |
